# Pain and Medical Research

**Published:** 1912-10-19

**Authors:** Herbert Snow


*To the Editor of* The Hospital.

### Pain and Medical Research.

*To the Editor of* The Hospital.

Sir,—May I ask you to favour me by correcting an entirely unwarranted and unwarrantable statement which appeared in your issue of September 14, and which has only just been brought to my notice?

This statement appears in the review of a work, "For and Against Experiments on Animals," by Mr. Stephen Paget, Hon. Secretary Research Defence Society. The words are: "Dr. Snow would allow experiments on animals to try new drugs and methods." It would seem to have been made on the authority of that work. It is wholly untrue both in substance and in fact. I should be very sorry to "allow" anything of the kind, considering no attitude possible to any medical practitioner who has dispassionately studied the subject of living-animal experimentation, cursorily known as "Vivisection," but that of total legislative prohibition.

Without at all going into the question of the horrible cruelties which have been and are continually perpetrated upon the lower animals under the false pretence of scientific investigation, a doctor who even cursorily examines the arguments upon which the practice of vivisection is commended for public support discovers at once that animal-experiment has never led to the smallest increase in medical knowledge or slightest improvement in medical practice; that all claims to that effect are perfectly spurious and easily proved to be so; that irrefutable scientific reasons preclude any and every argument based on phenomena in the sub-human animals and applied to man; that in very numerous instances—in fact, whenever reliance has been placed on such arguments—the profession has been grossly misled, and has been forced to confess the error, usually not until many valuable lives had been sacrificed.

The doctor aforesaid promptly finds, as I did some eight years ago, that the practice of vivisection rests solely upon a commercial basis, and is supported only by such methods or arguments as would commend themselves to an unscrupulous manufacturer pushing a lucrative trade. Very large capital is involved in the maintenance of the vivisection system. Were that not so, it could hardly stand its ground for a day. The "science" involved is no more than trade bluff.

I will add that I learnt this only when what was almost an accident led to my appearing as a witness before the late Royal Commission on Vivisection to give evidence on the bogus cancer researches, which are now confessed to be futile as regards the human species. I knew them to be worthless. On all other topics included in the wide domain of vivisection-investigation I was absolutely ignorant, as are some 999 out of every 1,000 medical practitioners. This led me to study the vivisection question, and ever since there has been no more uncompromising advocate of total prohibition. The fact is universally known among all who have any dealings with vivisection, and hence I say that such an assertion as the above—apparently not made by your reviewer, but put forth on the authority of the book he is considering—is inexcusable.

Trusting you will be able to find space for this correction.—Yours faithfully,

Herbert Snow, M.D. (Lond.)

